# Ações educacionais da Planificação da Atenção à Saúde: percepções dos profissionais em duas Regiões de Saúde do Brasil

**DOI:** 10.1590/0102-311XPT177724

**Published:** 2025-04-25

**Authors:** Sofia Guerra, Pedro Marques, Ana Coelho de Albuquerque, Isabella Samico, Luciana Santos Dubeux, Eronildo Felisberto, Diogenes Ferreira dos Passos, Marina Ferreira de Medeiros Mendes, Camila Soares de Vasconcelos

**Affiliations:** 1 Grupo de Estudos em Gestão e Avaliação em Saúde, Instituto de Medicina Integral Prof. Fernando Figueira, Recife, Brasil.; 2 Instituto Aggeu Magalhães, Fundação Oswaldo Cruz, Recife, Brasil.; 3 Universidade Rural de Pernambuco, Recife, Brasil.

**Keywords:** Capacitação de Recursos Humanos em Saúde, Avaliação em Saúde, Educação Interprofissional, Aprendizagem, Transferência de Treinamento, Health Human Resource Training, Health Evaluation, Interprofessional Education, Learning, Training Transfer, Capacitación de Recursos Humanos en Salud, Evaluación en Salud, Educación Interprofesional, Aprendizaje, Transferencia de Entrenamiento

## Abstract

A Planificação da Atenção à Saúde (PAS) visa apoiar estados e municípios na reorganização das Redes de Atenção à Saúde em seus territórios, oferecendo um conjunto de ações educacionais, em forma de oficinas, tutorias e cursos. Este artigo analisa as ações educacionais da PAS em duas Regiões de Saúde do Brasil, uma no Nordeste (região A) e uma no Norte (região B). A pesquisa abrangeu profissionais de saúde e tutores da PAS, totalizando 17 entrevistados. Os dados foram coletados por meio de entrevistas individuais semiestruturadas, que buscavam compreender as opiniões sobre aprendizagem, mudanças de comportamento, dificuldades e sugestões de melhoria. As entrevistas foram gravadas, transcritas e submetidas à análise temática de conteúdo. As categorias de análise foram elencadas de acordo com o Modelo Integrado de Avaliação do Impacto do Treinamento. Os resultados indicaram a aquisição de conhecimentos, habilidades e atitudes relacionados aos processos de territorialização; classificação de risco familiar; estratificação de risco populacional; manejo de usuários com doenças crônicas, gestantes e crianças menores de dois anos; planejamento, monitoramento e avaliação; entre outros. Houve importantes mudanças de comportamento, com melhoria da comunicação, colaboração e satisfação profissional. As principais dificuldades referem-se à resistência dos profissionais; à implementação do bloco de horas; ao uso e preenchimento de instrumentos; e ao acesso à atenção especializada. As principais sugestões foram a continuidade das ações educacionais presenciais, com participação dos facilitadores e gestores locais, e a garantia do horário protegido para dedicação às ações educacionais.

## Abstract (en)

Healthcare Planning (HP) aims to support states and municipalities in the reorganization of healthcare networks in their territories, offering a set of educational actions, in the form of workshops, tutorials and courses. This article analyzes the educational actions of HP in two Health Regions of Brazil, one in the Northeast (region A) and one in the North (region B). The survey covered health professionals and HP tutors, totaling 17 interviewees. Data were collected via individual semi-structured interviews, which sought to understand opinions about learning, behavior changes, difficulties and suggestions for improvement. The interviews were recorded, transcribed and submitted to thematic content analysis. The analysis categories were listed according to the integrated training impact evaluation model. Results indicated the acquisition of knowledge, skills and attitudes related to the processes of territorialization; family risk classification; population risk stratification; management of patients with chronic diseases, pregnant women and children under two years; planning, monitoring and evaluation; among others. There were important changes in behavior, with improved communication, collaboration, and job satisfaction. The main difficulties refer to the resistance of professionals; the implementation of block scheduling; the use and responses to instruments, and access to specialized care. The main suggestions were the continuity of face-to-face educational actions with the participation of local facilitators and managers, and the guarantee of protected time dedicated to educational actions.

## Resumen

La Planificación de la Atención Sanitaria (PAS) tiene como objetivo apoyar a los estados y municipios en la reorganización de las redes de atención a la salud en sus territorios al ofertar un conjunto de acciones educativas mediante talleres, tutorías y cursos. Este artículo analiza las acciones educativas de la PAS en dos Regiones Sanitarias de Brasil, una en el Nordeste (región A) y otra en el Norte (región B). En el estudio participaron profesionales de la salud y tutores de PAS, con un total de 17 encuestados. Los datos se recopilaron mediante entrevistas individuales semiestructuradas, que buscaron identificar las opiniones sobre el aprendizaje, los cambios de comportamiento, las dificultades y las sugerencias de mejora. Las entrevistas fueron grabadas, transcritas y sometidas a análisis de contenido temático. Las categorías de análisis se enumeraron según el modelo integrado de evaluación de impacto de la capacitación. Los resultados indicaron, entre otros aspectos, la adquisición de conocimientos, habilidades y actitudes relacionadas con los procesos de territorialización; la clasificación del riesgo familiar; la estratificación del riesgo poblacional; el manejo de usuarios con enfermedades crónicas, embarazadas y niños menores de 2 años; la planificación, seguimiento y evaluación. Hubo cambios importantes en el comportamiento, con una mejor comunicación, colaboración y satisfacción profesional. Las principales dificultades se refirieron a la resistencia de los profesionales; a la implementación del bloque de horas; al uso y finalización de instrumentos; y al acceso a atención especializada. Las principales sugerencias fueron la continuidad de las acciones educativas presenciales con la participación de elementos que facilitan y de gestores locales; y la garantía del horario programado para la dedicación a acciones educativas.

## Introdução

O Sistema Único de Saúde (SUS) encontra-se na sua terceira década de existência, superando muitas adversidades e acumulando experiências exitosas ^1^ ^,^ ^2^. No entanto, persistem desafios na consolidação das Redes de Atenção à Saúde (RAS), visando reduzir a fragmentação da assistência, considerando as diferentes realidades, as necessidades de saúde da população e a dimensão e complexidade do território brasileiro ^3^.

A estruturação das RAS é essencial para consolidar os princípios do SUS, sobretudo da equidade, integralidade e universalidade. A atenção primária à saúde (APS) tem papel central nessa organização, devendo desempenhar com eficiência quatro atributos principais: (i) acesso de primeiro contato dos usuários com o sistema de saúde; (ii) longitudinalidade, mantendo uma fonte continuada de atenção; (iii) integralidade, mantendo uma diversidade de serviços disponíveis para que o usuário receba atenção integral; e (iv) coordenação da atenção, integrando o cuidado que o paciente recebe em outros serviços ^4^. A Atenção Especializada (AE) deve apoiar a APS, em um sistema de cuidados integrais, cujo cuidado é centrado nas necessidades de saúde das pessoas e no cuidado ao usuário e favorece a atuação interprofissional, interdisciplinar e integrada das diferentes equipes e serviços ^5^.

Em consonância com a proposta das RAS, a Planificação da Atenção à Saúde (PAS) é uma estratégia de gestão e organização que promove a educação permanente em saúde dos profissionais, visando ao desenvolvimento de competências para organização dos processos de trabalho da APS e AE com foco nas necessidades dos usuários ^5^. Entre outras atividades estruturantes, a PAS oferece um conjunto de ações educacionais em formato de oficinas teóricas, tutorias e cursos de curta duração, visando à aquisição dos conhecimentos, habilidades e atitudes necessários à reorganização dos trabalhos e à melhoria da articulação entre níveis ^6^ ^,^ ^7^ ^,^ ^8^.

Estratégias de educação permanente em saúde são importantes para reorientar a atuação profissional, com ênfase na abordagem integral do processo saúde-doença, na valorização da APS e da interprofissionalidade e no fortalecimento do SUS. É nesse contexto que a planificação se insere, promovendo uma aprendizagem significativa, incentivando a reflexão crítica, a autogestão, a colaboração em equipe e a transformação das práticas no local de trabalho ^9^. 

A aprendizagem é uma mudança relativamente permanente no comportamento, com desenvolvimento dos conhecimentos, habilidades e atitudes. Porém, ainda não está claro como a aprendizagem e as competências se desenvolvem ao longo do tempo a partir de um treinamento ^10^. Com investimentos crescentes em treinamento, desenvolvimento e educação profissional, é necessário investigar os resultados dessas ações e seus benefícios aos serviços ^11^. Estudos sobre treinamento, desenvolvimento e educação profissional podem determinar até que ponto as práticas de ensino alcançam os resultados esperados, identificando aspectos facilitadores e dificultadores, que auxiliam na identificação e correção de deficiências nas ações educacionais, melhorando o apoio organizacional à formação e à aplicação de novos conhecimentos no trabalho ^12^ ^,^ ^13^.

A maior parte das pesquisas sobre treinamento, desenvolvimento e educação profissional tem focado na aprendizagem, na transferência (ou aplicação prática) do treinamento e seus impactos no trabalho, visto que os benefícios de qualquer ação educacional são determinados não apenas pela aprendizagem, mas também pela capacidade de aplicar esse conhecimento, reconhecendo-se a influência do ambiente e do contexto na produção dos resultados ^14^. É necessário analisar as ações de treinamento, desenvolvimento e educação profissional não apenas pelos benefícios aos participantes, mas também pela eficácia das equipes e organizações ^15^.

Este artigo analisa as ações educacionais da planificação na perspectiva dos profissionais em duas Regiões de Saúde do Brasil. Espera-se compreender as reflexões dos participantes sobre os conhecimentos, habilidades e atitudes adquiridos, identificar os desafios enfrentados e as sugestões de melhoria, de forma a orientar futuras estratégias de treinamento, desenvolvimento e educação profissional no SUS.

## Métodos

Trata-se de um estudo avaliativo, de abordagem qualitativa, que integra a pesquisa *Efetividade da Estratégia de Planificação da Atenção à Saúde em Quatro Regiões de Saúde no Brasil* - Pesquisa EfetivaPAS ^16^. O estudo em tela foi realizado em duas Regiões de Saúde do Brasil, uma no Nordeste (região A) e outra no Norte (região B), e utilizou elementos conceituais descritos por Abbad ^17^ sobre avaliação de treinamentos e na modelização das ações educacionais da estratégia PAS previamente elaborada ^18^.

As ações educacionais da planificação visam melhorar a capacidade do sistema de saúde por meio do desenvolvimento de conhecimentos, habilidades e atitudes necessários à reorganização dos processos de trabalho e à articulação das RAS ^6^ ^,^ ^18^. Consistem em oficinas, tutorias e capacitações, envolvendo profissionais de saúde, técnicos e gestores. São utilizadas práticas problematizadoras, focando na organização dos processos de trabalho e na coordenação entre níveis, promovendo reflexão crítica e o planejamento de ações concretas para a melhoria dos serviços de saúde ^6^ ^,^ ^8^. São desenvolvidas de maneira simultânea e integrada nas unidades da APS e da AE, em três etapas: (1) ciclos mensais de oficinas, tutorias e organização dos processos, com duração mínima de 12 meses; (2) supervisão das unidades para aperfeiçoamento dos processos; e (3) novos ciclos de oficinas e tutorias, conforme necessidades identificadas na etapa anterior ^6^. 

As oficinas apresentam o arcabouço teórico da planificação, podendo ser customizadas pelas regiões, abordando temas como RAS, a APS, territorialização, vigilância em saúde, organização da atenção à saúde, abordagem familiar, assistência farmacêutica, sistemas de informação em saúde, sistemas logísticos, de apoio diagnóstico e de monitoramento, e contratualização das equipes. A tutoria acontece dentro dos serviços, promovendo desenvolvimento contínuo de conhecimentos, habilidades e atitudes, apoio direto aos profissionais, reflexão sobre a prática, elaboração de planos de ação para avaliação contínua e identificação de fragilidades ^6^. É importante destacar que, durante o planejamento das ações educacionais, previa-se a realização das tutorias em formato presencial. Entretanto, em virtude da pandemia de COVID-19 que ocorreu no Brasil no primeiro trimestre de 2020, as tutorias passaram a ser realizadas em formato virtual, devido à necessidade de reorganizar os serviços para a assistência da população acometida por COVID-19 ^19^.

A definição das regiões de estudo foi realizada pelo Conselho Nacional de Secretários de Saúde (CONASS), de maneira intencional, tendo como critério a continuidade no desenvolvimento da planificação nas Regiões de Saúde. Em cada região incluiu-se um município com unidades laboratório da planificação. Participaram profissionais das unidades da APS e tutores da planificação em ambas as regiões do estudo, totalizando 17 entrevistados (Tabela 1). 

**Tabela 1:** Caracterização dos entrevistados por Região de Saúde. Brasil, 2022.

Categoria dos entrevistados	Região A	Região B	Total
Profissional da APS	4	5	9
Tutor da unidade de APS	3	2	5
Tutor municipal	1	-	1
Tutor regional	1	1	2
Total	9	8	17

APS: atenção primária à saúde.

Fonte: elaboração própria.

Devido à especificidade das informações buscadas, os entrevistados também foram selecionados intencionalmente, considerando seu papel na planificação, sua participação nas ações educacionais e suas responsabilidades na implementação das atividades propostas (Quadro 1). A coleta de dados ocorreu entre maio e setembro de 2022, por meio de entrevistas individuais semiestruturadas, com duração média de 60 minutos. As entrevistas foram realizadas presencialmente nas unidades de APS, com agendamento prévio e consentimento dos informantes. Não houve recusas.

**Quadro 1:** Responsabilidades e competências dos entrevistados na estratégia de Planificação da Atenção à Saúde (PAS).

CATEGORIA DOS ENTREVISTADOS	RESPONSABILIDADES E COMPETÊNCIAS
Tutores regionais	- Participação nas oficinas e tutorias nas unidades laboratório dos municípios-polo e no ambulatório de especialidades. - Condução das oficinas teóricas regionais. - Tutoria mensal nas unidades laboratório dos demais municípios-polo. - Supervisão do processo e apoio aos tutores municipais e à equipe do ambulatório de especialidades no período de dispersão. - Elaboração geral da linha de base do projeto. - Registro geral, monitoramento e avaliação do projeto.
Tutores municipais	- Participação nas oficinas e tutorias nas unidades laboratório dos municípios-polo. - Condução na organização dos macroprocessos na unidade laboratório do próprio município. - Tutoria das demais unidades municipais na fase de expansão. - Colaboração na elaboração da linha de base do projeto. - Registro, monitoramento e avaliação do projeto no município.
Tutores das unidades de APS	- Participação nas oficinas e tutorias nas unidades laboratório. - Condução na organização dos macroprocessos nas unidades. - Colaboração na elaboração da linha de base do projeto. - Registro, monitoramento e avaliação do desenvolvimento do projeto nas unidades.
Profissionais de saúde	- Participação nas oficinas e tutorias nas unidades laboratório. - Colaboração na organização dos processos de trabalho nas unidades. - Colaboração no registro, monitoramento e avaliação do desenvolvimento do projeto nas unidades.

APS: atenção primária à saúde.

Fonte: adaptado de Conselho Nacional de Secretários de Saúde ^6^.

Os roteiros semiestruturados buscavam compreender a opinião dos informantes sobre a aprendizagem, mudanças de comportamento e atitudes após o treinamento, dificuldades na transferência do treinamento e sugestões de melhoria. As entrevistas foram gravadas e transcritas na íntegra, submetidas à análise temática de conteúdo ^20^. As categorias de análise foram elencadas de acordo com o Modelo Integrado de Avaliação do Impacto do Treinamento (IMPACT, acrônimo em inglês) ^17^ (Quadro 2).

**Quadro 2:** Categorias de análise das ações educacionais da Planificação da Atenção à Saúde (PAS).

CATEGORIAS DE ANÁLISE	DEFINIÇÃO
Aquisição de conhecimentos, habilidades e atitudes	Percepção dos participantes sobre os conhecimentos, habilidades e atitudes adquiridos por meio do treinamento
Mudanças de comportamento	Percepção dos efeitos de treinamentos no comportamento dos egressos
Dificuldades na transferência do treinamento	Dificuldades na aplicação bem-sucedida dos conhecimentos, habilidades e atitudes adquiridos durante o treinamento
Sugestões para melhorar a aprendizagem e a transferência do treinamento	Sugestões dos entrevistados para melhorar a aprendizagem e a retenção dos novos conhecimentos, habilidades e atitudes e para a aplicação prática desses conhecimentos no dia a dia dos serviços

Fonte: adaptado de Araújo et al. ^32^.

**Figura 1:**
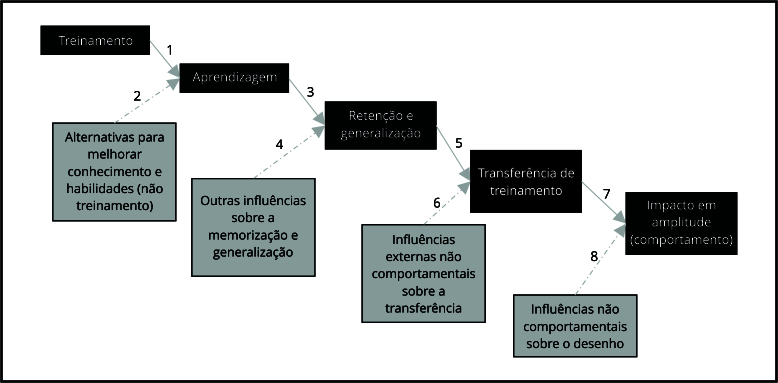
Representação esquemática das relações entre treinamento e resultados.

As categorias analíticas baseiam-se nas relações de causalidade entre elas: um treinamento eficaz promove aprendizagem (aquisição de novos conhecimentos, habilidades e atitudes), retenção do conteúdo (memorização e generalização), aplicação dos conhecimentos, habilidades e atitudes no trabalho (transferência de treinamento) e mudanças de comportamento (impacto em amplitude) (Figura 1).

Fonte: adaptado de Zerbini et al. ^33^. Nota: seta contínua: relação de causalidade entre as categorias analíticas; seta pontilhada: demais fatores de influência.

A Pesquisa EfetivaPAS foi aprovada pelo Comitê de Ética em Pesquisa do Instituto de Medicina Integral Prof. Fernando Figueira (parecer CAEE: 34198320.5.0000.5201).

## Resultados e discussão

### Aquisição de conhecimentos, habilidades e atitudes

Acerca da aquisição de conhecimentos, habilidades e atitudes, em ambas as regiões, os profissionais mencionaram vários macro e microprocessos básicos da APS, como territorialização, cadastramento das famílias, classificação de risco familiar, diagnóstico local e estratificação de risco populacional. A compreensão mais profunda do território e da população adscrita permite que os profissionais adequem as intervenções em saúde, elaborem planos de cuidados mais eficazes e ofereçam uma assistência mais qualificada aos usuários ^21^. A territorialização é essencial para uma atuação mais eficaz da APS, promovendo uma abordagem centrada no usuário e adaptada ao contexto local ^22^ ^,^ ^23^.

“*Com a territorialização, tivemos que realmente conhecer os limites da nossa área. Tivemos uma noção, tanto geográfica como social, de toda área. Em uma mesma área há diferenças sociais. Algumas coisas que tivemos que adaptar: as visitas, o acesso à área, o nosso tratamento a esse usuário. Muitas vezes os meios estão influenciando algumas patologias, e a gente entendendo o meio social e o território, tem como elaborar um plano de cuidado melhor*” (RA4, profissional).

Os profissionais também relataram novos conhecimentos sobre o cuidado de usuários crônicos, gestantes e crianças menores de dois anos. Reconheceu-se a importância do olhar ampliado durante visitas domiciliares, não se limitando ao usuário, mas buscando compreender as demandas da família e a manutenção do cadastro atualizado dos pacientes, o que facilita o acompanhamento e a prestação de um cuidado mais efetivo ^21^. A aprendizagem desses macroprocessos tem ampliado a capacidade da APS de identificar problemas e realizar encaminhamentos baseados em critérios objetivos. Estudos ^22^ ^,^ ^24^ ^,^ ^25^ têm apontado mudanças no perfil dos usuários encaminhados após a qualificação da APS para os processos de cadastramento, diagnóstico local e estratificação de risco, com redução de encaminhamentos incorretos.

Na região A, os profissionais de saúde entrevistados destacaram a aquisição de conhecimentos sobre o acompanhamento odontológico de gestantes e crianças menores de dois anos, ressaltando que essa prática, tradicionalmente, não é rotina nos serviços públicos. Após a discussão desse tema na planificação, segundo os entrevistados, a APS da região A aumentou significativamente sua oferta de atendimentos odontológicos para essa população, superando a quantidade de atendimentos realizados pela rede privada da região.

Sobre os macroprocessos de programação e monitoramento, a construção e o uso de instrumentos informatizados, como planilhas e checklists, permitiram um melhor planejamento da oferta de serviços, estabelecimento de metas, monitoramento e avaliação periódica dos processos de trabalho, com a identificação de pontos de melhoria. Esses instrumentos estão intimamente relacionados ao conhecimento do território, planejamento das ações e visitas domiciliares, contribuindo para uma prestação de serviços mais eficiente e direcionada às reais necessidades da população ^22^ ^,^ ^26^.

A organização dos agendamentos, fluxos internos de atendimento e processos de trabalho também foi importante, permitindo um melhor planejamento das atividades e gerenciamento da agenda dos profissionais. Com a implementação do bloco de horas, as consultas individuais passaram a ser agendadas previamente no horário específico escolhido pelo usuário, de acordo com a sua disponibilidade, eliminando a necessidade de chegar cedo e aguardar por longos períodos nas unidades de saúde ^23^ ^,^ ^27^. Para os profissionais entrevistados, o horário protegido proporcionou tempo suficiente para atender aos agendamentos e às demandas espontâneas, participar de reuniões de planejamento, discussões de caso e resolver questões administrativas: “*Não tínhamos o hábito de agendar por telefone, de atender demanda espontânea. Hoje, a gente consegue fazer o bloco de horas, e está funcionando. Tinha tumulto na unidade; hoje, cada paciente tem seu horário*” (RB7, profissional).

Os profissionais da região A ressaltaram a importância da classificação de risco durante a pandemia de COVID-19, destacando que aprender a determinar a gravidade dos casos de síndrome gripal e outros problemas de saúde trouxe mais clareza quanto ao direcionamento dos pacientes: “*Só o fato de entendermos que classificar o risco com segurança é importante, quando chegava a síndrome gripal a gente ia para a classificação, e só encaminhava quem precisava ser encaminhado*” (RA14, tutor). Do mesmo modo, em Uberlândia, Minas Gerais, a classificação de risco, elaborada durante a planificação, foi fundamental para a tomada de decisões clínicas e orientação dos profissionais de saúde, em um período de grande incerteza e demanda elevada, especialmente para a população idosa do município ^25^.

Sobre a atenção às condições crônicas, destacou-se o aprendizado referente à avaliação e ao manejo desses usuários, com a construção coletiva de novas diretrizes clínicas para uma gestão assertiva e de qualidade: “*Em relação às diretrizes clínicas, o manejo atualizado de algumas condições crônicas em situações específicas fez com que conseguíssemos manejar melhor alguns perfis de paciente*” (RA17, profissional). Assim como em estudos semelhantes ^22^ ^,^ ^24^, a qualificação do cuidado ofertado aos usuários com doenças crônicas foi considerada uma das maiores conquistas da planificação.

Além da reorganização dos processos de trabalho nas unidades, em ambas as regiões, os entrevistados relataram a aquisição de conhecimentos sobre as funções e os atributos da APS e AE no âmbito das RAS, a importância da comunicação, do compartilhamento do cuidado e da continuidade assistencial entre níveis.

“*Eu não esperava essa complexidade toda, não imaginava que iria trabalhar com toda uma rede. Imaginava que ia trabalhar somente com APS, fazer pré-natal, consulta de hanseníase, malária. Na verdade, é um serviço muito mais amplo, que tira a APS da base de uma pirâmide - que era assim que eu me via - para uma melhoria na rede. ‘Esse paciente é meu, esse paciente é da especializada, essa paciente é do CAPS’. Todos eles podem ir para esses serviços, mas eles não vão deixar de ser meus, eles moram no meu território; independente do risco deles, são da APS*” (RA18, tutor).

Esses resultados estão em consonância com achados anteriores ^23^ ^,^ ^26^, em que a Planificação, enquanto instrumento de gestão e organização, não só provocou mudanças nos processos de trabalho, mas permitiu a reflexão dos gestores, profissionais e usuários sobre os problemas a serem enfrentados e as mudanças necessárias. A aquisição desses conhecimentos fortalece a APS como elemento chave na constituição das RAS e instrumento fundamental para melhoria do acesso, integralidade e longitudinalidade do cuidado.

### Mudanças de comportamento

Quanto às mudanças percebidas no comportamento dos profissionais, em ambas as regiões, os entrevistados foram unânimes em relatar uma reação inicial de medo e resistência às ações educacionais, associando a planificação ao aumento de trabalho, reforçando achados anteriores ^28^. A mudança de percepção foi gradual, com o entendimento de que a planificação é uma estratégia para a qualificação profissional e reorganização dos processos de trabalho já existentes, seguindo um processo lógico bem estabelecido ^6^ ^,^ ^26^.

“*Sempre imaginei que era mais trabalho, mais um programa que inventam para ter mais papel para preencher. Mas quando comecei a participar, na verdade, percebi que é troca de experiência, conversa, melhoramento daquilo que já está sendo feito*” (RA18, tutor).

Outras mudanças unânimes foram as transformações positivas nas relações interpessoais. Destacam-se a melhoria da comunicação entre os profissionais; a valorização do trabalho multidisciplinar e interprofissional; um sentimento de pertencimento e importância dentro da equipe; maior abertura ao diálogo e colaboração; maior união da equipe e satisfação profissional. Outros estudos ^26^ ^,^ ^28^ também evidenciaram sentimentos de união, valorização e reconhecimento profissional na planificação.

“*Entendemos a importância de cada categoria profissional dentro da APS. Não dá para fazer APS sem ACS* [agente comunitário de saúde]*, sem a enfermagem, sem o médico, sem o NASF* [Núcleo Ampliado de Saúde da Família]*, sem a equipe multi. Cada um se viu muito importante para aquele objetivo ser alcançado. O vigia, na questão do acolhimento; a recepção com o fluxo interno da UBS* [unidade básica de saúde]*. Não é só o profissional que está dentro do consultório, é todo mundo igual. A equipe se uniu*” (RA14, tutor).

Também houve mudanças significativas na recepção e no acolhimento dos usuários, com desenvolvimento de habilidades de comunicação, escuta mais qualificada e maior preocupação com o acesso aos serviços e exames. Os profissionais passaram a se comunicar com mais gentileza e empatia, além de tomar medidas proativas, como monitorar agendamentos e realizar busca ativa dos faltosos: “*O atendimento, a maneira de atender, de receber o usuário. Principalmente essa questão de não deixar o paciente sem resolver o problema dele*” (RB6, tutor). O estudo de Costa ^27^, em Goiás, evidenciou mudanças de comportamento semelhantes.

Ademais, especificamente na região A, os entrevistados apontaram mudanças de comportamento e atitudes relacionadas ao maior comprometimento com responsabilidades profissionais; maior adesão aos protocolos clínicos; e uma postura mais crítica dos profissionais, questionando encaminhamentos inadequados, falta de retorno sobre usuários e falhas na obtenção de medicamentos.

### Dificuldades na transferência do treinamento

A principal dificuldade na transferência dos conhecimentos obtidos para a prática foi a resistência inicial dos profissionais, seja pela não aceitação da nova dinâmica de funcionamento, seja por questões atitudinais, de relacionamento da equipe, ou pelo adoecimento laboral relacionado ao enfrentamento da pandemia. Muitos profissionais não acreditavam na capacidade de implementar os novos macroprocessos, reagindo negativamente diante da complexidade de algumas tarefas. Médicos mostraram particular resistência, especialmente em relação ao cumprimento da carga horária de trabalho contratualizada, no atendimento de demandas espontâneas, além de relutância em participar de tarefas administrativas, preferindo atividades diretas de atendimento.

Apesar do sucesso do bloco de horas na maioria dos serviços, dificuldades persistiram devido à grande demanda de usuários, que apresentavam resistência em chegar no horário marcado, além de relutância de profissionais em cumprir sua carga horária. Experiências anteriores ^8^ ^,^ ^28^ de planificação indicaram que as Regiões de Saúde não tinham autonomia na contratação, lotação e aumento da carga horária de seus profissionais, dificultando a ampliação do horário de funcionamento das unidades, a implementação do bloco de horas e a proteção da carga horária para dedicação às atividades da planificação. Diversos movimentos de sensibilização e alinhamento com a gestão e com os profissionais de saúde foram necessários para avançar nessas questões. 

Outra dificuldade foi a compreensão, o preenchimento e o uso das planilhas e dos instrumentos de planejamento e monitoramento. Apesar dos esforços para fornecer orientação e esclarecer dúvidas, alguns profissionais ainda apresentavam dificuldades no manuseio desses instrumentos. A pandemia de COVID-19 levou à descontinuidade dos processos de cadastramento dos usuários e ao uso dessas ferramentas, exigindo esforços adicionais para retomar essas práticas. Ainda, há que se lidar com a instabilidade dos sistemas, especialmente do cadastro, causando inconsistências que afetam a confiabilidade dos dados.

“*Alguns instrumentos de reconhecimento de risco familiar, a gente está retomando. A maioria dos instrumentos foram quebrados pela pandemia, estamos resgatando agora. Mas algumas coisas são bem complicadas de entender, principalmente o preenchimento da planilha e o reconhecimento da área. Os ACS têm muita dificuldade com o cadastro, é muita gente que muda, o sistema é muito frágil, tem muito problema. A ACS diz que fez o cadastro, quando você vai ver, não está no cadastro*” (RB6, tutor).

Ferramentas informatizadas são importantes para a melhoria dos registros clínicos, a integração entre níveis de atenção e a gestão de cuidados longitudinais. No entanto, a implementação dessas ferramentas é complexa, requer investimentos das diferentes esferas governamentais, desenvolvimento de software adequado, infraestrutura apropriada (computadores, conexão à internet) e, sobretudo, capacitação dos profissionais ^22^.

O distanciamento social necessário ao enfrentamento da pandemia também interrompeu algumas atividades regulares da equipe, como as tutorias presenciais, a discussão de casos, grupos terapêuticos e visitas domiciliares: “*Tínhamos horários protegidos para reuniões. Paramos por causa da pandemia. Não fazíamos mais reuniões em equipe toda. Pararam as visitas domiciliares e as reuniões dos Hiperdia*” (RB7, profissional).

Evidências científicas já elucidaram ^21^ ^,^ ^22^ ^,^ ^24^ ^,^ ^27^ a influência da planificação na qualificação do cuidado, na melhoria do acesso aos serviços e na redução da fixação do usuário na AE sem justificativa clínica (efeito velcro). Entretanto, os profissionais ainda relataram dificuldades no encaminhamento à AE, especialmente para algumas especialidades que só podem ser agendadas via Sistema de Regulação (SISREG), resultando em atrasos nas consultas e nos tratamentos. Essas dificuldades são exacerbadas pela insuficiência de especialistas na rede: “*Temos dificuldade atualmente em encaminhar para pediatra e obstetra. A unidade especializada está com déficit, não conseguimos agendar, precisamos de mais profissionais*” (RB1, tutor). Ainda, a contrarreferência dos usuários à APS é problemática, mesmo quando o retorno é realizado pelas unidades de AE que participaram da planificação, devido à ausência de transferência de informação, prejudicando o acompanhamento e a continuidade do tratamento.

“*A contrarreferência é mais complicada, não temos retorno. Aqui na UBS, o ACS consegue fazer um retorno do que aconteceu depois que o paciente tem alta. Mas não temos nenhum protocolo, relatório ou documento do que foi feito na AE para nós. Não há contrarreferência desses pacientes, e o tempo de demora para acesso à AE é outro desafio*” (RA17, profissional).

A diminuta oferta de AE do SUS é bem evidenciada na literatura. Uma das causas é a insuficiência de aporte financeiro para a provisão desses profissionais ^8^. Outra questão é que, historicamente, o cuidado médico especializado no Brasil tem sido guiado pelos interesses das corporações médicas e das regras do mercado, inibindo a estruturação desses serviços no SUS ^29^. Experiências anteriores de planificação ^24^ relacionaram a insuficiência de vagas na AE à pouca integração entre níveis de atenção e Regiões de Saúde, à inexistência de protocolos padronizados com critérios de encaminhamento e à presença de serviços especializados de acesso aberto no território, operando sem articulação clara com a RAS.

O acesso à AE também se relaciona com a regulação assistencial. Centrais de regulação e complexos reguladores são vistos tanto como espaços privilegiados de gestão no SUS quanto como estruturas que se distanciam das necessidades do território e tornam-se barreiras de acesso aos serviços ^29^ ^,^ ^30^. No caso da planificação, esses sistemas precisam ser readequados para operar conforme a nova lógica proposta ^8^, dialogando com mecanismos de coordenação assistencial construídos coletivamente pelos profissionais: novos protocolos, diretrizes clínicas, plano de cuidado compartilhado entre níveis, interconsulta, grupos de trabalho interdisciplinar (discussões de casos, regulação negociada), microrregulação, entre outros.

Na planificação do Distrito Federal, a reorganização dos processos regulatórios resultou na criação do Complexo Regulador em Saúde (CRDF), que passou a regular o acesso aos serviços conforme os novos protocolos, a classificação de risco e os demais critérios de priorização definidos e pactuados coletivamente durante a planificação. A reorganização da regulação e a criação do CRDF foram relacionadas à redução das filas de espera para consultas e exames e à organização e transparência na oferta de AE na região ^24^.

Espera-se que, com a instituição da Política Nacional de Atenção Especializada, seja possível melhorar a regulação e o acesso à AE no SUS, estruturando esses serviços especializados em articulação com a APS e complexos reguladores. A oferta e regulação do acesso a exames, as consultas e os procedimentos especializados passarão a ser programados de maneira integrada, garantindo às equipes de APS máxima autonomia na tomada de decisões ^5^.

A sobrecarga de trabalho e a rotatividade profissional também foram fatores dificultadores da aplicação dos conhecimentos aprendidos, devido ao tempo e esforços necessários para ensinar novos profissionais e lidar com a alta demanda de usuários. Os recém-chegados têm dificuldade de adaptação à rotina de trabalho da unidade planificada, e a falta de tempo para capacitação contribui para o desconforto e a desorientação. A rotatividade sazonal de médicos e enfermeiros, particularmente em fevereiro (concursos, admissão em cursos de residência), agrava essa situação, exigindo esforços adicionais para integrar e capacitar novos profissionais.

A proposta de organização do processo de trabalho, integração das equipes e qualificação profissional da planificação por si só é uma potencialidade. Entretanto, requer sucessivos momentos de diálogo e envolvimento dos profissionais para reforço das responsabilidades ^28^, o que demanda tempo. Ainda, a rotatividade profissional nos municípios é contextualizada pelos desafios impostos à APS no SUS: subfinanciamento, falta de prioridade política, desvalorização dos profissionais e precarização dos vínculos trabalhistas. Essas dificuldades impactam diretamente na integralidade e longitudinalidade dos cuidados, destacando a necessidade de fortalecimento das estratégias de educação permanente, como a planificação, e a criação de mecanismos de valorização e apoio à fixação dos profissionais nos territórios ^21^ ^,^ ^26^.

Na região A, uma dificuldade específica foi a resistência dos usuários à territorialização, não aceitando mudar sua UBS de referência: “*Para a população, mudar de um sistema que já estavam acostumados há décadas. A dificuldade maior foi fazer com que as pessoas se adaptassem ao novo processo de atenção*” (RA3, profissional). Ainda na região A, questões relacionadas à infraestrutura e tecnologia prejudicaram o atendimento odontológico: “*Na odontologia, minha maior dificuldade é em relação à estrutura. Não poder fazer o que tem que ser feito com o que tenho fisicamente. Às vezes são coisas simples, como uma luz funcionando*” (RA12, profissional). A dependência desses recursos tecnológicos para procedimentos básicos ressalta a importância de infraestrutura funcional para a prestação de serviços de saúde adequados, sendo necessário mobilizar gestores para melhorar os investimentos nas regiões de saúde ^28^.

Na região B, a complexidade de organizar um atendimento multidisciplinar coordenado foi uma dificuldade relatada. Apesar dos esforços, a experiência não teve sucesso devido à incompatibilidade das agendas dos profissionais envolvidos: “*O atendimento em circuito não deu certo. Fizemos uma vez o plano de cuidado, mas não conseguimos mais porque precisa da agenda dos outros profissionais, médico, enfermeiro, do NASF - psicóloga, fisio, nutri*” (RB6, tutor). 

A compatibilização das agendas dos profissionais é essencial para a realização do circuito de atendimento, que consiste em consultas sequenciais do usuário com todos os profissionais envolvidos no seu cuidado ^6^. O plano de cuidados compartilhado, quando corretamente utilizado, possibilita o acesso às informações clínicas dos usuários nos diversos pontos da RAS. A construção coletiva dos planos permite que os profissionais desenvolvam uma visão ampliada das necessidades do usuário, alcançando consensos sobre as intervenções necessárias; estimula o comprometimento dos profissionais com as ações a serem realizadas conjuntamente para alcance dos objetivos. No entanto, a efetividade dos planos compartilhados depende do compromisso dos profissionais, da compatibilização das agendas e da comunicação adequada para gerir problemas, decisões e conflitos ^31^.

### Sugestões para melhorar a aprendizagem e a transferência do treinamento

No tocante às sugestões para facilitar a aprendizagem e melhorar a aplicação dos conhecimentos na prática, os entrevistados enfatizaram a continuidade das ações educacionais da planificação. Sugeriu-se um novo ciclo de oficinas teóricas para resgate conceitual e a criação de um calendário anual dos momentos formativos, com menor intervalo entre os encontros, proporcionando suporte contínuo aos profissionais. O resgate teórico e a proximidade dos encontros seriam importantes para minimizar os problemas oriundos da resistência e rotatividade, facilitando o engajamento dos recém-chegados e daqueles que não puderam participar anteriormente.

“*É fundamental um resgate com todos, igual era feito no começo. Na realidade, uma educação continuada. A cada dois meses eles* [os facilitadores] *vinham. Quando já estávamos querendo dar um passo atrás, vinha tutoria e resgatava tudo o que já tínhamos feito e propunha coisas novas. Era um apoio extra. A diretora que trabalha comigo não participou, não entende, não concorda. Mas é porque esses profissionais não participaram desde o início. Santo de casa não faz milagre*” (RB6, tutor).

Além da manutenção e periodicidade das ações educacionais, destacou-se a importância da participação de facilitadores e supervisores estaduais e municipais nesses momentos, proporcionando suporte ao trabalho desenvolvido pelos tutores das unidades. A presença de atores externos parece suscitar mudanças, fortalecer o processo e garantir maior adesão dos profissionais ^28^.

“*A nossa tutoria funciona muito mais quando tem o facilitador aqui, presencial, com toda a equipe. A prata da casa geralmente não brilha muito, por mais que a gente tenha consolidado esse posto de tutor, mas a presença do facilitador do CONASS ajuda muito, parece que amarra o processo, e é isso que a gente está sentindo falta*” (RB6, tutor).

Em ambas as regiões, pontuou-se a necessidade de conferir dinamicidade às oficinas e tutorias, com metodologias ativas que incentivem a participação, como rodas de conversa, questionamentos, exemplos práticos e oportunidades para a aplicação do conhecimento adquirido. Os profissionais relataram preferir tutorias às oficinas, devido ao enfoque não só na aprendizagem, mas na aplicação prática dos conhecimentos.

Em ambas as regiões, pontuou-se a importância do horário protegido para dedicação à planificação (reuniões, estudos e discussões em equipe). O horário protegido permite reuniões mais frequentes, favorecendo a assimilação do conteúdo, transferência do treinamento e condução de mudanças gradativas nas unidades ^27^. Ressaltou-se a necessidade de ampliar o quadro de recursos humanos, permitindo que os profissionais se dediquem efetivamente à aplicação prática dos conhecimentos adquiridos, sem prejudicar o trabalho assistencial.

As tutorias, originalmente programadas para serem presenciais, foram adaptadas para o formato virtual em virtude das restrições impostas pela pandemia de COVID-19. Com a normalização das operações nas unidades de APS da região A, profissionais de saúde sugeriram o retorno ao formato presencial das tutorias, argumentando que esse método potencializa o aprendizado ^19^. Além disso, sugeriram tutorias focadas em um único tema, com carga horária ampliada para no mínimo dois dias, possibilitando tempo suficiente para discussão e implementação das melhorias propostas, evitando excesso de informações e desvio dos temas, especialmente porque o tempo disponível é limitado. Um resumo dos achados encontra-se disposto no Quadro 3.

**Quadro 3:** Similaridades e especificidades acerca da aprendizagem e aspectos relacionados em duas Regiões de Saúde do Brasil.

CATEGORIA DE ANÁLISE	SIMILARIDADES ENTRE AS REGIÕES DE SAÚDE	ESPECIFICIDADES DAS REGIÕES DE SAÚDE
Aquisição de conhecimentos, habilidades e atitudes	Aquisição de conhecimentos relativos: - Aos macro e microprocessos básicos da APS, como a territorialização, o cadastramento das famílias, a classificação de risco familiar, o diagnóstico local e a estratificação de risco populacional. - À compreensão mais profunda do território e da população adscrita; - à identificação das subpopulações alvo por fator de risco ou condições de saúde. - Ao manejo de usuários crônicos, gestantes e crianças. - Ao uso de diretrizes clínicas atualizadas. - Ao manuseio de instrumentos informatizados, como planilhas e checklists, permitindo melhor planejamento e organização dos serviços. - À organização dos agendamentos por meio do bloco de horas, com melhor gerenciamento do tempo dos profissionais. - À organização dos fluxos internos de atendimento das unidades. - À humanização e qualificação do atendimento; com melhoria da recepção e do acolhimento dos usuários. - À compreensão das funções e dos atributos da APS e da RAS. - À valorização do trabalho interprofissional e atendimento multidisciplinar. - À comunicação entre níveis e compartilhamento do cuidado.	Região A: - Melhorias no acompanhamento odontológico de gestantes, crianças e usuários com doenças crônicas. - Importância da classificação de risco e manejo adequado dos pacientes durante a pandemia de COVID-19.
Mudanças de comportamento	- Reação inicial de medo e resistência à planificação, com adesão gradual, a partir da visualização de mudanças positivas nos serviços. - Maior abertura à comunicação e colaboração nas relações profissionais. - Sentimento pertencimento e união da equipe. - Satisfação profissional. - Postura mais cordial e acolhedora no atendimento aos usuários. - Maior proatividade no cuidado aos usuários.	Região A: - Maior comprometimento com as responsabilidades profissionais. - Maior adesão aos protocolos clínicos. - Cuidado centrado nas necessidades dos usuários. - Postura mais crítica dos profissionais para com a organização da RAS.
Dificuldades na transferência do treinamento	- Resistências profissionais à reorganização dos processos de trabalho. - Alguns profissionais com perfil inadequado para o trabalho na APS. - Resistências à implantação do bloco de horas e ao cumprimento da carga horária exigida nas unidades, especialmente entre a categoria médica. - Dificuldades na compreensão dos instrumentos de planejamento e monitoramento. - Descontinuidade dos processos durante a pandemia de COVID-19. - Sobrecarga de trabalho. - Rotatividade profissional. - Longos tempos de espera para consulta com as especialidades. - Entraves na contrarreferência e transferência da informação clínica dos usuários.	Região A: - Resistência dos usuários à mudança de unidade de referência. - Dificuldade na elaboração dos planos de cuidado para idosos frágeis. - Insuficiência de equipamentos e insumos adequados para o atendimento odontológico. Região B: - Dificuldades na realização do circuito atendimento multidisciplinar, devido à agenda dos profissionais. - Dificuldades em trabalhar a promoção da saúde com os usuários.
Sugestões para melhorar a aprendizagem e a transferência do treinamento	- Continuidade das ações educacionais. - Realização de um novo ciclo de oficinas teóricas para resgate conceitual e sensibilização dos profissionais. - Criação de um calendário formal dos momentos formativos, com periodicidade menor à atual. - Necessidade da presença dos facilitadores e dos tutores estaduais, regionais e municipais, como importante fator de mobilização dos esforços. - Conferir dinamicidade às ações educacionais, com maior uso de metodologias ativas de aprendizagem. - Garantia do horário protegido para participação nas ações educacionais e para estudo e dedicação à implementação dos processos. - Ampliação dos recursos humanos nas unidades, de forma a garantir o horário protegido e não comprometer os atendimentos.	Região A: - Tornar as tutorias menos cansativas, com a discussão de um único tema, em maior profundidade. - Aumento da carga horária das tutorias. - Realização das tutorias em formato presencial.

APS: atenção primária à saúde; RAS: Redes de Atenção à Saúde.

Fonte: elaboração própria.

Este estudo apresenta algumas limitações, inerentes ao tratamento qualitativo dos dados, buscando compreender a aprendizagem e os aspectos relacionados à planificação por meio da subjetividade dos atores. Outra limitação é a impossibilidade de extrapolação dos resultados para realidades distintas das regiões estudadas. Além disso, para este artigo, não se contemplou a perspectiva de outros atores envolvidos na planificação, como facilitadores institucionais, gestores e usuários. Por outro lado, privilegiou-se o enfoque dos profissionais e tutores diretamente envolvidos com o cuidado à saúde na APS. Futuros artigos que contemplem demais perspectivas podem fornecer informações importantes sobre o processo de planificação e ampliar as reflexões presentes neste estudo.

## Considerações finais

Os resultados desta pesquisa evidenciam que a planificação é um potente dispositivo de gestão, organização das RAS e, sobretudo, de qualificação profissional. Apesar das dificuldades, os profissionais de saúde relataram aquisições significativas de conhecimentos, habilidades e atitudes essenciais à qualificação da APS. A planificação propiciou momentos valiosos de discussão do trabalho realizado pela APS, qualificando processos como territorialização, cadastramento, classificação de risco familiar, diagnóstico local, estratificação de risco populacional, recepção e acolhimento dos usuários, gestão das condições crônicas de saúde, programação e monitoramento das ações e dos serviços, organização dos atendimentos e da oferta de serviços das unidades, entre outros.

Além da reorganização dos processos de trabalho, a planificação possibilitou transformações positivas no comportamento dos profissionais, com maior abertura ao diálogo e ao trabalho em equipe, e maior comprometimento com as responsabilidades. Como consequência, diversos efeitos positivos foram visualizados pelos profissionais, incluindo um planejamento mais eficaz das ações e serviços, manejo mais adequado das condições de saúde, aprimoramento dos registros informatizados, melhoria do acesso às consultas e aos exames, aumento da satisfação dos profissionais e dos usuários, entre outros. 

Entretanto, ainda restam lacunas a serem preenchidas. O uso correto das ferramentas informatizadas, a redução do tempo de espera para atendimento especializado, a melhoria da contrarreferência, a sobrecarga de trabalho, a alta rotatividade de profissionais, a infraestrutura insuficiente e a necessidade de proteção da carga horária dos profissionais para dedicação às atividades da planificação são alguns dos problemas evidenciados neste estudo.

Para a consolidação das RAS no SUS, é essencial reconhecer e valorizar a APS como coordenadora do cuidado e ordenadora da rede. A planificação é uma estratégia que estimula essa compreensão e visa melhorar a articulação e integração dos serviços. Contudo, melhorias efetivas nos sistemas de saúde exigem movimentos contínuos de sensibilização, responsabilização e envolvimento dos interessados. É necessário que estados, municípios e regiões de saúde possam dar continuidade às discussões iniciadas durante a planificação, endossando investimentos em estrutura, recursos humanos e estratégias de educação permanente em saúde, para que as dificuldades apontadas neste estudo possam ser minimizadas. Tais iniciativas são fundamentais para a superação do modelo biomédico assistencial e para a construção de serviços compatíveis com as necessidades de saúde da população.
